# Deeds and Words: Farmers’ Attitude-Paradox in Collective Action for Small-Scale Irrigation

**DOI:** 10.3390/ijerph20010549

**Published:** 2022-12-29

**Authors:** Shanshan Miao, Xueqin Zhu, Wim Heijman, Zengwei Xu, Qian Lu

**Affiliations:** 1 Development and Research Institute of Central Jiangsu, Yangzhou University, Yangzhou 225009, China; 2 Environmental Economics and Natural Resources Group, Wageningen University, 6708 WG Wageningen, The Netherlands; 3 Agricultural Economics and Rural Policy Group, Wageningen University, 6708 WG Wageningen, The Netherlands; 4 Department of Economics, Czech University of Life Sciences Prague, 165 00 Prague, Czech Republic; 5 Business School, Yangzhou University, Yangzhou 225127, China; 6 Department of Economics and Management, Northwest A&F University, Xianyang 712100, China

**Keywords:** collective action, attitude–behavior paradox, income heterogeneity, social ties, instrumental variable probit model

## Abstract

We explore the mechanisms of the attitude–behavior paradox and how multiple stakeholders strategize to compromise their attitudes and behaviors. Through an instrumental variable probit model, we examine the effect of income heterogeneity and social ties on the farmers’ attitude–behavior paradox for collective action. The empirical results demonstrate that weak and strong ties, income heterogeneity, interaction terms, education, community environment, and community rules negatively affect the paradox, whereas water stealing and water use conflicts positively affect it. After dividing the paradox into two forms, we find that weak ties, the interaction terms thereof, negatively affect the paradox for “having negative attitude but do have behavior”, while income heterogeneity negatively affects the paradox for “having positive attitude but no behavior”. We contribute to the understanding of mechanisms whereby economic incentives and social structures interplay in addressing the above paradox. We conclude by discussing the implications for policies to overcome this social dilemma.

## 1. Introduction

Small-scale irrigation facilities’ collective action is important to increase agricultural output and improve rural households’ welfare. However, initiating and sustaining collective action is difficult, as farmers’ positive attitudes often fail to transform into real collective action participative behavior [1,2,3]. Or rather, farmers’ cooperative behavior is weakly related to their attitude [4]. This inconsistency between farmers’ expressed intentions or willingness and their lack of action, or the reverse, is called the attitude–behavior paradox [5]. The paradox leads to reduced efficiency in resource extraction and maintenance, as well as a deficit incentive for sustainable common-pool resource management [6,7,8]. The following questions then arise: What leads to this farmers’ attitude–behavior paradox? Which factors could reconcile this paradox?

Previous studies utilized planned behavior theory, attribution theory, value–belief-norm theory and the cognitive dissonance theory [9,10,11,12,13] to explore the individual’s attitude–behavior paradox in the domains of sustainable tourism, environment, consumption, safety and study behavior, etc. [14,15,16,17,18]. Although many variables have been identified to explain individuals’ attitude–behavior paradox from the socio-psychological perspective, e.g., other people’s behaviors [19,20], personal capabilities [21], social norms, perceived behavior control [17], awareness of consequence and ascription of responsibility [22], due to the complexity of human decisions and their interactions with social and economic institutions, heterogeneity is rarely concerned. Heterogeneity plays a significant role in addressing social dilemmas [23], which could be an innovative theoretical perspective for illuminating farmers’ attitude–behavior paradox relating to collective action in small-scale irrigation facilities.

Addressing farmers’ attitude–behavior paradox is important, as it “can have important social and ecological consequences and understanding both its nature and effects can help in neutralizing the negative and enhancing the positive” [24]. To achieve this, one concern relates to how versatile stakeholders interact and their strategies to balance self-interests against collective welfare, that is, understanding the mechanism of farmers’ attitude–behavior paradox. The other concern lies in how economic and social heterogeneity deciphers the attitude–behavior paradox by changing attitude or promoting behaviors [25]. Therefore, there is a need to understand different paradoxes from a socio-economic perspective by employing individual and collective utility strategies.

This paper aims to understand farmers’ attitude–behavior paradox and examine the effects of income heterogeneity and social ties in addressing this paradox by employing an instrumental variable probit (IV probit) model. By identifying different forms of attitude–behavior paradoxes, our study seeks to ascertain not only the total effects of income heterogeneity and social ties on farmers’ paradox. We also explore the separate effects of whether income heterogeneity addresses this paradox for “having positive attitude but no behavior” or whether the social ties addresses the farmers’ paradox for “having negative attitude but do have behavior.”

The remaining paper is structured as follows: Section 2 explores the mechanisms of farmers’ attitude–behavior paradox, Section 3 explains the theoretical and conceptual framework, Section 4 describes the study area and methods, Section 5 presents the empirical results, Section 6 discusses the results, and Section 7 concludes the study.

## 2. Mechanisms of Farmers’ Attitude–Behavior Paradox

The provision of small-scale irrigation facilities often demands substantial investment. However, the farming system in the Northern China is characterized as a smallholder farming system. Without large stakeholders being involved, smallholders simply cannot afford such a huge investment. Collective action for small-scale irrigation facilities has been promoted by national governments and international organizations. Despite the advantages of collective action, in providing public goods, and fostering community development, the initiation and sustenance of collective action faces formidable challenges, that is, the farmers’ attitude–behavior paradox toward collective action.

Understanding how stakeholders frame their decision-making process for collective action is a prerequisite for deciphering the farmers’ attitude–behavior paradox. As the main participants, multiple types of stakeholders interact in common-pool resource management settings, and they hold competing or conflicting attitudes owing to individual endowment characteristics, information asymmetry, and confined social interactions. The paradox originates from the fact that an individual’s utility-maximizing strategy does not automatically lead to an optimal collective welfare optimum—that is, “the individual utility vs. collective welfare dilemma” [4].

Therefore, integrating individual and collective utility is the key to address farmers’ attitude–behavior paradox. Collective action is assumed to take place and sustain under circumstances where public interests can fit with private interests. To find a compromise between farmers’ attitude and behavior lies in how versatile stakeholders with different endowments are embedded in social structures, and how incentives and information interact to strike a balance between private and collective interests to achieve desired outcomes.

To reveal the mechanisms of the attitude–behavior paradox, allowing for sub-divisions would be a better approach to understand these differences. We thus divided farmers’ attitude–behavior paradox into two forms: farmers who have a positive attitude but not the desired behavior and farmers who have a negative attitude but do have the desired behavior.

### 2.1. Farmers Who “Have Positive Attitude but No Behavior”

For those farmers who have a positive attitude but not the desired behavior, decisions against collective action represent a social dilemma situation [26]. For farmers to exhibit the desired behavior depends on whether it will enable them to simultaneously “get their share of the accepted collective goal and to maximize individual utility” [4]. Previous studies demonstrate that actors with larger endowments who are proponents of shared resources usually act as “elites” and play an active role in the initiation of collective action [27]. In this sense, heterogeneity is crucial in addressing the social dilemmas of collective action [23]. However, many studies have shown that economic heterogeneity is multi-faceted; different aspects of heterogeneity may affect different types of collective actions and these effects are moderated by complex contextual factors. In this sense, income heterogeneity can be taken as an important variable that may influence stakeholders’ ability and/or incentive to undertake the initiatives of collective action, that is, material payoffs will stimulate them to act in a collective-oriented way, thus generating motivations for altering behaviors [7,28].

### 2.2. Farmers Who Have Negative Attitude but Do Have Behavior

The structural effects of social behavior highlight the role of social ties in motivating knowledge sharing, resource mobilization, and making mutually advantageous decisions in collective action [29,30,31]. When individuals’ participating behavior engaging in collective action is contingent upon others, how each participant triggers others until the chain reaction reaches the threshold to initiate collective action is the key to overcome this type of attitude–behavior paradox. The outcomes depend on the network of social ties that channel the chain reactions [32]. Regarding the homogeneous or heterogeneous contentions between weak ties and strong ties [33], some scholars maintain that both social ties are important [34,35], while others argue that weak ties are more effective than strong ones in predicting desired outcomes [33,36]. In fact, social ties influence farmers’ attitude–behavior paradox in different ways—being behavior-directed or attitude-directed, depending on the structural properties of social ties (weak ties or strong ties) as well as stakeholders’ strategies, searching for individual utility maximization and/or collective welfare maximization [37,38]. Stakeholders could alter their behavior or attitude by embedding in the structure of social interactions to understand what strategy best satisfies their interests, or strong ties may have direct influence to change an individual’s interests and internal motivations toward participation through social pressures [39,40].

## 3. Theoretical Framework

**Figure 1:**
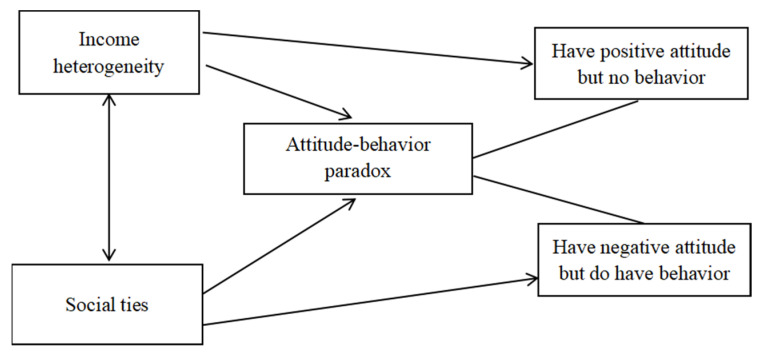
Conceptual framework of farmers’ attitude–behavior paradox.

Based on the analysis above, overcoming social dilemmas and sustaining the collective action of small-scale irrigation facilities depend on how to reconcile farmers’ attitude–behavior paradox; income heterogeneity and social ties are potentially transformative ingredients in altering the farmers’ attitude–behavior paradox. Figure 1 demonstrates this relationship between the dependent and independent variables and the hypothesized signs of these relationships.

Income heterogeneity is often perceived to influence collective action in situations where stakeholders may or may not have a common interest in regulating the use of common-pool resources [41]. Income heterogeneity is defined as the earnings inequality across individuals and it is closely related to individuals’ motivation to overcome social dilemmas and contribution in collective action [42]. However, the effect of income heterogeneity is ambiguous, as income heterogeneity is often associated with the distribution of benefits from the management of a common-pool resource and the alternative income earning opportunities (e.g., off-farm income). For example, increasing inequality could stimulate major resource users to contribute for reaping more benefits and simultaneously encourage small users to free-ride [43], whereas alternative income earning opportunities outside common-pool resources may also reduce higher-income users’ incentive to participate in collective action [44]. To overcome social dilemmas, income heterogeneity is thus deemed effective in fostering actors who have commons interest to transform attitudes into behaviors, by gaining more of the positive externalities. Therefore, we hypothesize the following:

**H1a:**  *Income heterogeneity is negatively associated with farmers’ attitude–behavior paradox*.

Based on the rational choice theory, farmers’ decisions on whether to participate in collective action are a consequence of how they calculate the benefits and costs. For the farmers who have positive attitude but no behavior, expectations that the benefits they receive will exceed the costs they pay will motivate them to transform their attitudes into participative behavior. Income heterogeneity may thus lead farmers to behave differently to make decisions, depending on how strongly the economic incentives to make these material benefits transform into participative behavior. We argue that income heterogeneity explains a substantial portion of this type of paradox for farmers who have positive attitudes but no behavior. Hence, we propose the following hypothesis:

**H1b:**  *Income heterogeneity is negatively associated with paradox for farmers who have positive attitudes but no behavior*.

In addition to material benefits, farmers are also driven by solidarity benefits, including rewards such as socializing, congeniality, the sense of group membership and identification, status resulting from membership, fun and conviviality, and the maintenance of social distinctions [45]. To obtain these benefits, farmers may strategically adjust or frame their attitudes or behaviors by social ties through information exchange, knowledge sharing, and social learning [46]. Thus, they can be aligned to homogenous or heterogeneous ties to pursue desired outcomes of collective action [47]. Therefore, we propose the following hypothesis:

**H2a:**  *Social ties are negatively associated with farmers’ attitude–behavior paradox*.

The embeddedness of weak or strong ties in social structures can be regarded as a mechanism to encourage individuals to engage in frequent interactions and facilitate information circulation; it allows them to access collective action, and to influence, negotiate, and bargain to reach their desired objectives [48,49]. Therefore, farmers’ attitudes can be altered by weak ties to acquire potential gains from other socially distant actors and to take advantage of the benefits not available within a highly clustered network [50]. Strong ties have the advantage of high-quality information exchange. Social norms and sanctions aligning with public interests are then generated in this process, which, in turn, addresses this paradox for farmers who have negative attitude but do have behavior [51]. In general, to compromise this type of paradox, farmers adapt themselves to permeate outward and transform their local contexts into wider ones by enacting interactions of networks and meta-networks to alter attitudes [52,53]. Therefore, we hypothesize the following:

**H2b:**  *Social ties are negatively associated with farmers’ paradox for having negative attitude but do have behavior*.

Previous studies demonstrated that inequality generates distinct group identities such as social classes, increasing income heterogeneity is more likely to intensify social heterogeneity which affects cooperation [54]. As economic incentives “may favor behaviors that nevertheless do not occur unless information (social ties) makes individuals aware that the incentive is available” [22], we mainly focus on how strong ties and weak ties linked to income heterogeneity, and its influence on farmers’ attitude–behavior paradox. According to Briggs [55], social support is associated with strong ties, which tend to provide emotional and instrumental support for daily life. Networks composed of weak ties that offer social leverage help individuals to change their opportunity structure for advancement, both of these ties are beneficial for resources access, and these ties can cut across race, ethnicity, and/or social class [56]. However, compared to high-income farmers who have more connections with dispersed and heterogeneous social networks, low-income farmers are more inclined to be constrained by the homogeneity of the strong ties, the similar information and resources circulated, limiting the opportunities for circulation of information and access of resource. Therefore, we formulate the following hypothesis:

**H3:**  *The interaction terms of social ties and income heterogeneity are negatively related to farmers’ attitude–behavior paradox, the interaction term between weak ties and income heterogeneity is more effective compared to the interaction term between strong ties and income heterogeneity*.

## 4. Study Area and Methods

### 4.1. Study Area and Variables

A household survey was conducted based on a case study of the Jinghui qu irrigation systems in Shaanxi Province, China. Jinghui qu irrigation system is China’s first modern large-scale irrigation project, which covered 48 towns, 6 counties and 3 cities (Xi’an, Xianyang and Weinan) of 1,465,000 mu (97,666.7 hectares), which is assumed to be representative of irrigation in the northeast of China. A multistage sampling strategy was utilized to select sample households. In each city, two counties differing in geographical location were selected based on their respective position in upstream, middle stream and downstream, that is, Jingyang County, Sanyuan County, Gaoling County, Lintong County, and Yanliang County. Within each county, we applied a stratified random sampling method to select 3 townships, we then randomly selected from a census of villages in each township in the upper, middle, and lower reaches of the canal network within the Jinghui qu irrigation districts. Finally, we randomly selected 8–10 household heads in each village. After excluding those with incomplete responses from 350 copies of the survey, 308 valid questionnaires were selected.

The study area is located in semi-arid districts with a continental monsoon climate, with an average annual rainfall of 399 mm; this rainfall is mainly concentrated in summer and autumn. The uneven distribution of rainfall often resulted in droughts in the spring and summer. In order to combat the drought, farmers cooperate in the form of contributing money or labour for the construction and maintenance of small-scale irrigation facilities. The irrigated areas are flat, and within these areas, the soil is fertile. The main crops are wheat, corn, cotton, and vegetables. The survey contained information on social demographics, management conditions of irrigation, social capital, social ties, and farmers’ attitudes toward collective action for small-scale irrigation facilities. The order and wording of the questions were carefully designed to ensure unbiased identification. As “a weakness of much of this research is the distinction between what survey respondents say and what they actually do” [57], to account for this weakness and avoid social desirability bias, information on participation behavior was collected from the list of irrigation managing committee personnel after the survey interview.

### 4.2. Measures

#### 4.2.1. Income Heterogeneity

As inequality in wealth or income is often regarded as the main indicator of heterogeneity, and annual income is easily observed at the household level. The household’s relative income position is employed to measure income heterogeneity [58]:

(1) $$RI_{i}=\frac{x_{i}-X_{ave}}{X_{max}-X_{min}}$$

$RI_{i}\;$ is the household relative income position in the community, $x_{i}\;$ is household i’s annual income, and $X_{ave}$ is the average value of the survey data. $X_{min}$ is the minimum value of the survey data and $X_{max}$ is the maximum value of the survey data.

#### 4.2.2. Social Ties

Social networks can be divided based on their relational and structural characteristics that are attributed to relational (i.e., strong ties and weak ties) and structural aspects (i.e., core networks and periphery networks). Our paper mainly focuses on the relational aspect of social networks which were modeled as weak ties and strong ties; social ties represent the web of social relationships that farmers maintain, including both intimate relationships with family and close friends and more formal relationships with other individuals and groups. It is through this web of social ties that individuals can be socially “integrated” into the larger society in which they live. Therefore, social ties highlight the role of stakeholders’ embeddedness in bridging or bonding networks to compromise the attitude–behavior paradox. The social ties are, thus, an important force to update farmers’ attitudes toward behaviors.

Strong ties represent a dense network that can be delineated as tightly clustered relationships, e.g., kin, neighbors, and intimate friends. These ties could provide instrumental support for small loans, child caring, etc., for ensuring daily basic needs [50]. Weak ties can be characterized as the exchange of information and knowledge among remote connections [59]. Therefore, a strong tie is measured by “the number of people you could ask for help to cope with the inconvenience and difficulties in daily life [60].” A weak tie is measured by “the number of people outside your immediate family and close friends for contacts and acquaintances.” Both of them are measured on a five-point scale which takes the value 1 if the respondent replies one to three, 2 if they reply “four to seven,” 3 if “eight to eleven,” 4 if “twelve and fifteen,” and 5 if “16 and more”.

### 4.3. Control Variables

Contextual elements have been identified to analyze individuals’ attitude–behavior paradox [61,62]. Normally, contextual elements refer to attributes, institutional constraints, and regulatory institutions [63]. Therefore, the control variables we selected were farmers’ social demographics, rules, and institutions. Community rules and institutions were selected to reflect the attributes of the community atmosphere that influence behavior and attitude in human-interactive situations [64]. Previous studies have demonstrated that the sustainability of common-pool resource management is more inclined to occur in a community with shared norms and rules [65]. We thus selected past experiences of water stealing and water use conflicts to reflect the level of rules for managing common-pool resources, as the rules are critical for small-scale irrigation facility construction and maintenance, especially for the rules-in-use by the participants.

### 4.4. Methods

To evaluate the effects of social ties and income heterogeneity on farmers’ attitude–behavior paradox for small-scale irrigation facilities collective action, we estimate the following system of equations using a two-stage IV probit model [66]:

(2) $$Y_{i}=\alpha +\beta X_{i}+\chi SN_{i}+\gamma RI_{i}+\varepsilon_{i}$$

where $Y_{i}$ is the binary variable that indicates 1 for inconsistency between attitude and behavior and 0 for consistency between attitude and behavior. Attitude–behavior paradox is defined as farmers who have positive attitude but no behavior or farmers who have negative attitude but have behaviour. Attitude–behavior consistency is defined as farmers who have both attitude and behaviour or have negative attitude and no behavior. This variable was estimated by taking the absolute value of the difference between attitude and behavior. $X_{i}$ is the vector of variables controlling for socio-demographic variables. $SN_{i}$ represents social ties that have the potential for endogeneity. $RI_{i}$ is the relative income position for income heterogeneity. Because it is difficult to find an appropriate regression methodology that can evaluate binary or continuous variables with endogenous variables, a linear structure to the endogenous variable was used to estimate the model [67,68]. Following this structure, the second-stage model is taken, with $SN_{i}$ as the endogenous variable. The first stage of the IV probit model is as follows:

(3) $$SN_{i}=\delta +\psi C_{i}+\eta Z_{i}+v_{i}$$

where $C_{i}$ is a vector of exogenous variables and $Z_{i}$ is the vector of instruments, which are highly correlated with the social ties but not correlated with the error term $\varepsilon_{i}$. Under the assumption of heterogeneity, the instrument variable should predict social ties, but it should be uncorrelated with outcomes other than through social ties. If Cov($SN_{i}$,$\varepsilon_{i}$)≠0, social ties under the program are endogenous.

We use the bivariate probit model to test for the necessity of recruiting instrument variables. The bivariate probit model tests for the endogeneity of social ties variables to the outcome variable by estimating Cov($SN_{i}$,$\varepsilon_{i}$) ≠ 0 or Cov($SN_{i}$,$\varepsilon_{i}$) = 0. If Cov($SN_{i}$,$\varepsilon_{i}$) = 0, then we cannot reject the null of exogeneity, and the simple probit estimation would be sufficient to gauge the effect. However, if Cov($SN_{i}$,$\varepsilon_{i}$) ≠ 0, then the endogeneity conditions hold; the IV probit estimation can provide consistent estimates of the effect.

Equations (1) and (2) are the first-stage regressions for measuring the instruments of social ties as well as other control variables in the outcome equation. We thus developed three pairs of models.

Pair A is estimated with the bivariate probit model for strong and weak ties separately.

Pair B is estimated using the IV probit model for strong and weak ties separately.

Pair C is estimated using the IV probit model for strong ties × income heterogeneity and weak ties × income heterogeneity separately.

As Zhou et al. [69] have identified interaction effects between social capital and income disparity, the interaction item constructed here is to explore the joint effects of income heterogeneity and social ties on farmers’ attitude–behavior paradox.

### 4.5. Instrument Variable of Social Ties

Endogeneity of social ties has been validated by a vast amount of literature [70,71,72]. This is mainly because social ties and participation in collective action have reverse causality relationships. For example, farmers with voluminous social ties are more inclined to participate in collective action, while farmers participating in collective action benefit from information exchange and knowledge sharing as well as increased income levels, increasing their inclination for further participation. Moreover, it is difficult to gauge residual confounding, such as unobserved variables including personality and family background, which may also correlate with social ties, when it cannot be argued that explanatory variables are not correlated with the error term. Therefore, it is important to select a decent instrumental variable for two prerequisites to be satisfied: The instrumental variable should not be correlated with the endogenous dependent variable, and it should be correlated with the independent variable other than through other endogenous variables. Therefore, a valid instrumental variable in our case should be correlated with social ties, but have no direct effects on the attitude–behavior paradox other than through social ties. Thus, we used the following instrumental variable methods for parameter estimation:

The distance to the provincial or national highways. This variable was selected to explain the relationship between distance and social ties. In rural China, farmers’ houses tend to be located closer to transportation routes such as national roads, so the distance from provincial or highways is closely related to the distribution of housing density and thus affects farmers’ social ties. At the same time, distance from provincial or national highways does not directly affect collective action and satisfies exogeneity. The Spearman correlation demonstrates that distance is not correlated with farmers’ attitude–behavior paradox (Spearman’s ρ = −0.074; *p* = 0.195), but is correlated with social ties (Spearman’s ρ = 0.112; *p* = 0.049). The selection of the instrumental variable fits the criteria of relevance and exogeneity.

Whether family members have the experience of metropolitan city migrant working: The selection of the instrumental variable fits the criteria of relevance and exogeneity because family members’ work experience outside the home helps expand farmers’ own social ties, but it is reasonable to assume that family members’ experiences do not directly influence their own small-scale irrigation facilities collective action decision-making. Spearman correlation analysis indicates that migrant working experience was not significantly correlated with the attitude–behavior paradox (Spearman’s ρ = −0.045; *p* = 0.428), while it was significantly correlated with social ties (Spearman’s ρ = 0.194; *p* = 0.001).

## 5. Results

### 5.1. Total Effects of the Attitude–Behavior Paradox

Table 1 summarizes the descriptive statistics of the sample. About 64.94% of respondents were willing to participate in collective action for small-scale irrigation facilities; however, only 49.03% of respondents had real participative behavior. Further, 64.6% of respondents showed the attitude–behavior paradox. Among this group, 23.7% of respondents did not have the positive attitude, but did have real participative behavior, while 40.9% showed willingness toward collective action without any participative behavior. Of the respondents, 80.84% received primary and secondary education. The average cultivated area for each household was 3.517 acres. Past experiences of water stealing and water use conflicts are on a medium level, specifically 3.6 and 2.5, respectively, compared with the maximum level of 5. The mean value of weak ties and strong ties were 4.47 and 3.58, respectively. Respondents had a high evaluation of their community atmosphere, for which the mean value was above 3.6. The empirical results of the IV probit model, which addresses the potential endogeneity of social ties, are shown in Table 2.

To simplify the table, the results of the first step of the regression are not shown. In the first step of regression results in both of the instrumental variables of (1) distance to main provincial and national highways, and (2) Whether family members have the experience of metropolitan city migrant working have significant effects on social ties, indicating that both of these instrumental variables are highly associated with farmers’ social ties. The listing in the three columns allows for the comparison of the results between the bivariate probit model and IV probit model. In the first bivariate model, all household demographic controls are incorporated into the model, except for the instrumental variables. In the second specification, weak ties and strong ties are evaluated separately, for which instrumental variables are introduced into the IV probit model. For the third specification, the interaction effects of social ties and income heterogeneity are included. The results show that both of the instrumental variables have significant effects on social ties. Moreover, the F value of the first-stage regression is above 10, demonstrating that the weak instrument problem does not exist [73]. The Wald test of exogeneity shows a *p* value less than 0.1, implying that we can reject the null hypothesis of exogeneity at the 10% level of significance. Thus, the probit regression with endogenous regressor ensures the accuracy of gauging the effect of the social ties and income heterogeneity on farmers’ attitude–behavior paradox.

Income heterogeneity is negatively and significantly related to farmers’ attitude–behavior paradox at the 1% level of significance. Thus, H1a is proved. This result implies that income heterogeneity leads to a distinction in farmers’ motivation to participate in collective action. This finding is consistent with Ruttan [74], who argued that economic heterogeneity is positively related to the initiation of collective action. Furthermore, the IV probit regression of instrumental variables indicates that instrumental variable selection is appropriate for addressing farmers’ attitude–behavior paradox. Appendix A shows the first-stage probit regression result, which validates the social ties instruments.

The coefficients of weak ties (−1.885) and strong ties (−0.688) are negatively and significantly related to farmers’ attitude–behavior paradox. This result demonstrates that “information benefits are expected to travel over all bridges, strong or weak” [75]. The effect of strong ties in reducing farmers’ attitude–behavior paradox mainly occurs through a highly clustered network by inflicting a monitoring mechanism on opportunistic behavior. Weak ties are better predictors of farmers’ attitude–behavior paradox as they link socially distant actors to unique and non-redundant networks. Thus, negative attitude based on these weak or strong ties can be altered to act toward a desired collective action. Our finding is in accordance with Santos et al. [76], who contended that collective action is more likely in heterogeneous networks. Therefore, H2a was confirmed. However, the interaction term of the social ties and income heterogeneity does not pass the significance test.

Experience of water stealing and water use conflicts are positively and significantly associated with farmers’ attitude–behavior paradox. This demonstrates that rules and regulations are essential for addressing the attitude–behavior paradox in small-scale irrigation facilities’ collective action. It plays a prominent role in distributing the interests between heterogeneity and the farmers’ attitude–behavior paradox. This empirical result can be corroborated by Poteete and Ostrom [77], who argued that rules and principles are of crucial importance for a group to initiate and sustain collective action.

The community’s rules and atmosphere are negatively and significantly related to the farmers’ attitude–behavior paradox. Institutions related to rules and regulations produce a series of incentives and create new social and economic circumstances [78]; thus, when costs and benefits inherent in participation of collective action are fairly distributed due to the rules and regulations, farmers are more inclined to reconcile their attitude–behavior paradox [79]. Therefore, a community with clear rules and norms is more inclined to overcome social dilemmas in initiating and sustaining collective action.

### 5.2. Separate Effects of the Attitude–Behavior Paradox

Income heterogeneity and social ties have been identified as variables for reconciling farmers’ attitude–behavior paradox. More tailored policy interventions are needed to address farmers’ different forms of paradox. Therefore, we divide farmers’ attitude–behavior paradox into two forms—“having negative attitude but do have behavior” and “having positive attitude but no behavior”—to explore how income heterogeneity and social ties address farmers’ attitude–behavior paradox. The empirical results are listed in Table 3.

For the first type of paradox, that is, farmers with a negative attitude but demonstrating desired behavior, the weak ties exerts negative and significant effects in explaining this kind of inconsistency. Our findings highlight the role of actors embedded in weak ties, which could provide more opportunities for interests sharing, information circulation and upward mobility [80,81]. This result is consistent with Granovetter [82], who attached importance to the cohesive power weak ties exert in overcoming social dilemmas. Compared with redundant relationships and homogeneous networks of strong ties, weak ties are beneficial for providing new opportunities and bridging the boundaries of local clusters [32].

The interaction terms between weak ties and income heterogeneity, as well as strong ties and income heterogeneity, exert negative and significant effects on farmers who have negative attitude but do have behavior. The empirical results demonstrate that the structural characteristics of social ties (weak ties and strong ties) exert significant effects on the behavior of farmers. Farmers’ decisions are social processes which are affected by social structures [83]. How farmers’ income heterogeneity is distributed throughout strong ties and weak ties, and how farmers alter their attitudes, are affected by their strategy embedded into social structures, and is crucial for understanding farmers’ paradox.

Income heterogeneity has significantly negative effects on farmers’ paradox of “having positive attitude but no behavior.” This result demonstrates that income heterogeneity could promote economic incentives for those who have positive attitude but no behavior to transform their positive attitude into collective actions. Therefore, the key to overcoming the attitude–behavior paradox of this form is to generate sufficient economic incentives and aligning these interests to achieve consistency in collective action.

## 6. Discussion

Our study arises from the question “Why do farmers not fit their deeds to their words in the collective action for small-scale irrigation facilities?” To answer this question, we explored the mechanisms of how stakeholders interact in different forms of attitude–behavior paradoxes (having positive attitude but no behavior and having negative attitude but having behavior) and thereafter examined the effects of income heterogeneity and social ties on farmers’ attitude–behavior paradox. On the whole, the complexity of the attitude–behavior paradox lies not only in the heterogeneous interests of stakeholders, but also the social structures formulated by social ties to reinforce or attenuate this inconsistency. Therefore, to address the attitude–behavior paradox, one needs to understand how income heterogeneity and social ties separately and interactively played a role in transforming or adapting to behaviors.

The first key finding is that our study reveals the socio-economic nature of farmers’ decision-making processes. A theoretical framework that explores the origins of the paradox is necessary. Our findings demonstrate that individual–collective utility inconsistencies lead to farmers’ attitude–behavior paradox, given that “individual utility depends not only on individual well-being, but also the well-being of the community to which the individual belongs” [84]. Future work examining other domains of attitude–behavior paradoxes, individual–collective utility may serve as a useful theorem to further understand the causal factors and mechanisms that reconcile such a paradox in typical populations.

Another novel finding from our research is that income heterogeneity and social ties could reconcile farmers’ attitude–behavior paradox. Income heterogeneity and social ties work differently when deciphering farmers’ attitude–behavior paradox into “farmers who have positive attitude but no behavior” and “farmers who have negative attitude but do have behavior”. Accordingly, farmers’ attitude–behavior paradox could be altered by increasing economic incentives and by embedding into weak and strong ties for social support and social leverage, to integrate individual and collective utility. Moreover, our research also underlines the interaction effect of income heterogeneity and social ties, which may expand the potential limits of economic or social effects to address the attitude–behavior paradox.

## 7. Conclusions

We employed the IV probit model to examine the effects of social ties and income heterogeneity on farmers’ attitude–behavior paradox for the collective action of small-scale irrigation facilities in Northwest China. We found that the attitude–behavior paradox can be reconciled by income heterogeneity and social ties. Farmers can achieve attitude–behavior consistency that is conditioned by how individuals embed themselves into different social ties to align common-pool resources, thus to maximize and integrate individuals as well as collective utilities. Our findings also suggest that stressing behavior or attitude change, while ignoring rules and community environment, will lead to unintended consequences aggravating the attitude–behavior paradox. Strategies that compromise the attitude–behavior paradox should create circumstances of rules and institutions where public interests can be combined with private interests.

Our study contributes to the theoretical framework of collective action research and has implications for developing economic incentives and social structures interventions to address the attitude–behavior paradox. Our result is important not only for common-pool resource domains of collective actions and social dilemmas, but also for other complex contexts involving various stakeholders to cooperate and achieve desired outcomes. Insights from this research demonstrate important policy implications for overcoming the farmers’ attitude–behavior paradox. Understanding the mechanisms of farmers’ attitude–behavior paradox can help policymakers to effectively alleviate failures in collective action and understand the micro-foundations of individual choice-making processes to overcome social dilemmas.

First, interventions could be directed to enhance income heterogeneity to promote elites’ contribution by altering their behavior to gain additional benefits of collective action, which are crucial for developing countries due to the externalities of the common-pool resources and many stakeholders competing for those resources. Second, participatory and action-led values and conventions are emphasized to expand both the weak and strong ties, providing the opportunities for policy interventions to promote the initiatives of social groups, cooperatives, and networks that can serve as a channel for information exchange and social learning. Finally, stressing behavior or attitude change alone, while ignoring rules and the community atmosphere, will lead to unintended consequences that aggravate the attitude–behavior paradox. Therefore, externally imposed incentive-compatible mechanisms are necessary to enhance farmers’ cooperative choices.

One limitation of our research is that we mainly explored the farmers’ attitude–behavior paradox from a socioeconomic perspective. A focus only on socioeconomic variables may fail to reveal their relationship with individuals’ attitudes or behaviors, leading to biased results. Future studies that explore the identified relationships should consider potentially confounding variables, such as those independently related to attitudes or behaviors. Interdisciplinary research combining socioeconomic and psychological theories is a future direction.

## Figures and Tables

**Table 1:** Descriptive statistics of main variables.

Variable	Description	Minimum	Maximum	Mean (S.D.)	Expected Effects
Independent variables					
Attitude	Would you like to contribute labour or money in small-scale irrigation facilities collective action?	0	1	0.501	
Behavior	The real participating behavior collected from list of irrigation managing committee personnel	0	1	0.478	
Paradox	Paradox between attitude and behavior (paradox = 1, no paradox = 0)	0.00	1.00	0.6396 (0.4809)	
*Dependent variables*					
Weak ties	The number of people outside your immediate family and close friends for contacts and acquaintances (1 = 1–3; 2 = 4–7; 3 = 8–11; 4 = 12–15; 5 = above 16)	2.00	5.00	4.4740 (0.7716)	−
Strong ties	The number of people you could ask for help to cope with the inconvenience and difficulties in daily life (1 = 1–3; 2 = 4–7; 3 = 8–11; 4 = 12–15; 5 = above 16)	1.00	5.00	3.5812 (1.1052)	−
Income heterogeneity	Based on the computation	−0.37	0.73	−0.0080 (0.7160)	+/−
Cultivated area	Real cultivated area (mu)	0.00	11.00	3.5170 (2.0520)	+/−
Water stealing	The frequency of water stealing happens (1 = never; 2 = occasionally; 3 = sometimes; 4= frequently; 5 = always)	1.00	5.00	3.6100 (1.2081)	+/−
Water use conflict	The frequency of water-use conflicts (1 = never; 2 = occasionally; 3 = sometimes; 4 = frequently; 5= always)	1.00	5.00	2.5377 (1.0100)	+/−
Community rules	The village’s rules and regulations executive conditions (1 = very bad; 2 = bad; 3 = neither bad nor good; 4 = good; 5 = very good)	2.00	5.00	4.6753 (0.6135)	+/−
Community atmosphere	What is the community’s atmosphere? (1 = very bad; 2 = bad; 3 = neither bad nor good; 4 = good; 5 = very good)	1.00	4.00	3.6169 (0.5898)	+/−
Instrumental variable 1	The distance to the highway 1 = 1–2 km; 2 = 3–5 km; 3 = 6–8 km; 4 = 9–12 km; 5 = above 12 km	1.00	5.00	3.6331 (0.9088)	
Instrumental variable 2	Whether family members have the experience of metropolitan city migrant working (1 = work; 2 = no work)	0.00	1.00	0.3604 (0.4809)	

**Table 2:** Estimation results of farmer’s attitude–behavior paradox.

	Bivariate Model		IV Probit Model		IV Probit Model with Social Ties*Income Heterogeneity	
	Model 1	Model 2	Model 3	Model 4	Model 5	Model 6
Weak ties	−0.213 (0.176)		−1.885 * (1.143)		−0.321 (0.224)	
Strong ties		0.312 *** (0.111)		−0.688 ** (0.335)		0.289 *** (0.110)
Weak ties*income heterogeneity					4.026 (4.818)	
Strong ties*income heterogeneity						−0.041 (1.168)
Income heterogeneity	−1.862 *** (0.667)	−1.994 *** (0.702)	−2.073 *** (0.778)	−2.171 *** (0.836)	−19.956 (21.741)	−1.816 (4.154)
Education	−0.266 * (1.452)	−0.036 ** (0.059)	−0.109 (0.199)	−0.275 (0.181)	−0.240 (0.155)	−0.310 ** (0.153)
Cultivated area	−0.017 (0.057)	−0.036 (0.059)	−0.031 (0.066)	0.007 (0.073)	−0.040 (0.065)	−0.042 (0.060)
cultivation experience	0.006 (0.011)	0.006 (0.011)	0.019 (0.015)	−0.004 (0.013)	−0.005 (0.011)	−0.004 (0.011)
Water stealing	1.290 *** (0.147)	1.251 *** (0.138)	1.366 *** (0.159)	1.692 *** (0.218)	1.317 *** (0.144)	1.295 *** (0.143)
Water use conflicts	0.299 *** (0.112)	0.225 * (0.116)	0.367 *** (0.139)	0.354 ** (0.146)	0.293 ** (0.118)	0.225 * (0.116)
Community rules	−0.261 (0.240)	−0.455 ** (0.216)	0.813 (0.773)	−0.515 ** (0.263)	−0.135 (0.306)	−0.472 ** (0.223)
Community atmosphere	−0.314 (0.199)	−0.300 (0.208)	−0.456 * (0.248)	−0.699 ** (0.282)	−0.400 (0.238)	−0.282 (0.224)
Cons	−0.812 (1.302)	−1.483 (1.405)	1.066 (1.971)	1.927 (1.994)	−0.595 (1.382)	−1.364 (1.409)
Pseudo R-squared	0.5836	0.6006				
Log-likelihood	−83.826	−80.406				
F-statistics			11.59	13.15	1090.08	364.77
Wald test of exogeneity			Chi2(1) = 3.09 Prob > chi2 = 0.079	Chi2(1) = 13.11 Prob > chi2 = 0.0003	Chi2(1) = 1.10 Prob > chi2 = 0.2945	Chi2(1) = 0.75 Prob > chi2 = 0.3873
R-squared			0.2811	0.3249	0.9759	0.9313
Adj R-squared			0.2544	0.2998	0.9750	0.9287

Note: ***, **, and * denote significance at the 1%, 5%, and 10% levels, respectively.

**Table 3:** Comparison between the two forms of attitude–behavior paradox: “having negative attitude but have behavior ” and “having positive attitude but no behavior”.

	Having Negative Attitude but Have Behavior				Having Positive Attitude but No Behavior			
	IV Probit Model		IV Probit Model Social Ties*Income Heterogeneity		IV Probit Model		IV Probit Model Social Ties*Income Heterogeneity	
Weak ties	−2.781 * (1.657)				−1.340 (1.061)			
Strong ties		1.048 (1.011)				0.324 (0.736)		
Weak ties*income heterogeneity			−0.821 *** (0.241)				−0.241 (0.168)	
Strong ties*income heterogeneity				−1.145 *** (0.351)				−0.394 (0.267)
Income heterogeneity	−3.652 *** (1.370)	−3.633 *** (1.188)			−1.440 * (0.788)	−1.202 * (0.720)		
Education	−0.143 (0.369)	−0.438 * (0.244)	−0.523 ** (0.217)	−0.493 ** (0.218)	−0.139 (0.191)	−0.204 (0.178)	−0.249 (0.161)	−0.248 (0.162)
Cultivated area	0.018 (0.111)	0.060 (0.089)	0.063 (0.079)	0.054 (0.078)	−0.022 (0.066)	−0.048 (0.068)	−0.028 (0.063)	−0.024 (0.063)
Cultivation experience	0.041 (0.026)	0.021 (0.018)	0.014 (0.015)	0.015 (0.151)	0.008 (0.015)	0.002 (0.014)	−0.003 (0.012)	−0.003 (0.012)
Water stealing	1.567 *** (0.264)	1.002*** (0.357)	1.342 *** (0.186)	1.344 *** (0.187)	1.344 *** (0.162)	1.179 *** (0.256)	1.261 *** (0.148)	1.280 *** (0.150)
Water use conflicts	0.404 * (0.234)	0.330 * (0.189)	0.220 (0.146)	0.223 (0.146)	0.375 *** (0.145)	0.345 ** (0.171)	0.292 ** (0.124)	0.293 ** (0.124)
Community rules	1.014 (1.041)	−0.493 * (0.297)	−0.682 *** (0.246)	−0.610 ** (0.240)	0.807 (0.914)	−0.183 (0.310)	−0.317 (0.270)	−0.275 (0.270)
Community atmosphere	−0.459 (0.409)	−0.359 (0.323)	−0.305 (0.283)	−0.259 (0.282)	−0.412* (0.236)	−0.340 (0.219)	−0.360 (0.221)	−0.389 * (0.223)
Cons	2.240 (2.894)	−3.566 (3.392)	−0.306 (1.664)	−0.855 (1.658)	−1.417 (1.711)	−2.965 (3.216)	−1.255 (1.634)	−1.422 (1.626)
F-statistics	6.24	4.81	240.18	152.52	12.55	6.15	372.00	178.07
Wald test of exogeneity	Chi2(1) = 5.21 Prob > chi2 = 0.022	Chi2(1) = 1.26 Prob > chi2 = 0.2622	Chi2(1) = 0.12 Prob > chi2 = 0.7241	Chi2(1) = 1.08 Prob > chi2 = 0.2991	Chi2(1) = 1.10 Prob > chi2 = 0.2946	Chi2(1) = 0.00 Prob > chi2 = 0.9561	Chi2(1) = 0.13 Prob > chi2 = 0.7184	Chi2(1) = 0.21 Prob > chi2 = 0.6445
R-squared	0.2651	0.2176	0.9255	0.8875	0.3591	0.2153	0.9370	0.8769
Adj R-squared	0.2226	0.1724	0.9216	0.8817	0.3305	0.0.1803	0.9345	0.8720

Note: ***, **, and * denote significance at the 1%, 5%, and 10% levels, respectively.

## Data Availability

The data are contained within the article.

## Appendix A

**Table A1:** The first stage result of the IV Probit model.

	Dependent Variables			
	Weak Ties	Strong Ties	Weak Ties*Income Heterogeneity	Strong Ties*Income Heterogeneity
Instrumental variable 1	0.056 (0.043)	0.482 *** (0.060)	1.133 *** (0.023)	0.934 *** (0.019)
Instrumental variable 2	0.204 ** (0.082)	−0.107 (0.115)	0.403 *** (0.147)	0.089 (0.123)
Education	0.085 *** (0.049)	0.032 (0.069)	−0.008 (0.016)	−0.015 (0.013)
Cultivated area	−0.005 (0.019)	0.046 * (0.027)	0.004 (0.006)	−0.004 (0.005)
Cultivation experience	0.009 ** (0.004)	−0.003 (0.005)	−0.001 (0.001)	0.0004 (0.001)
Water stealing	0.035 (0.033)	0.264 *** (0.046)	−0.0007 (0.010)	0.017 * (0.009)
Water use conflicts	0.020 (0.040)	0.110 ** (0.056)	−0.013 (0.013)	−0.021 ** (0.010)
Income heterogeneity	−0.055 (0.224)	−0.102 (0.314)		
Community rules	0.602 *** (0.065)	−0.047 ** (0.092)	−0.056 *** (0.020)	−0.004 (0.017)
Community atmosphere	−0.058 (0.066)	−0.252 (0.093)	0.027 (0.021)	−0.050 *** (0.018)
Cons	1.005 ** (0.446)	1.607 ** (0.627)	0.219 (0.136)	0.228 ** (0.114)
R-squared	0.281	0.307	0.934	0.929
F-statistics	11.59	13.15	465.33	430.10

Note: ***, **, * denote 1%, 5%, 10% of significance level, respectively.
